# Social Media–Based Cancer Education: Bibliometric and Thematic Analysis

**DOI:** 10.2196/77214

**Published:** 2025-10-06

**Authors:** Yang Xie, Zhenning Guo, Xiangning Zeng, Denghui Zhai, Gaoqiang Zhai, Yinzhou Feng, Huang Huang

**Affiliations:** 1 Department of Urology, The Second Nanning People's Hospital, The Third Affiliated Hospital of Guangxi Medical University, Nanning, China; 2 School of Foreign Studies, China University of Petroleum (East China), Qingdao, China; 3 Department of Thyroid and Breast Surgery, The Second People's Hospital of Qinzhou, Qinzhou, China; 4 Department of Pharmacy, Guangxi Hospital Division of The First Affiliated Hospital, Sun Yat-sen University, Nanning, China; 5 Department of Urology, Guangxi Hospital Division of The First Affiliated Hospital, Sun Yat-sen University, Nanning, China; 6 College of Computer Science and Technology, Qingdao Institute of Software, China University of Petroleum (East China), Qingdao, China; 7 Department of Neurosurgery, Guangxi Hospital Division of The First Affiliated Hospital, Sun Yat-sen University, 3 Foziling Road, Qingxiu District, Nanning, 530022, China, 86 15274504689

**Keywords:** social media platforms, bibliometric analysis, health literacy enhancement, digital health technology applications, cancer education

## Abstract

**Background:**

Traditional education for patients with cancer faces challenges related to timeliness, accessibility, and a personalized approach. Social media has emerged as a novel platform for delivering cancer-related educational content, garnering growing academic interest. However, a comprehensive assessment of the current research landscape in this domain is lacking.

**Objective:**

This study aimed to identify research hotspots; trace the evolution of social media–based education for patients with cancer; and map the leading journals, institutions, and international collaboration networks in this field.

**Methods:**

A bibliometric and thematic analysis was conducted using tools, such as VOSviewer, Bibliometrix, and CiteSpace, to examine articles indexed in the Web of Science Core Collection from 2011 to 2025. The analysis explored publication trends, author and institutional collaboration networks, keyword co-occurrence, factor analysis, thematic clusters, and the evolution of disciplinary keyword categories.

**Results:**

A total of 119 publications were retrieved. The Journal of Medical Internet Research was the most productive journal in this field, publishing 13 articles (10.9%). The University of Minnesota was the most productive institution, contributing 6 publications (5.0%). The United States accounted for the largest proportion of publications (56/119, 47.1%), with 5 of the top 10 institutions based in the country. The United States also led the international collaboration network. Keyword analysis identified key research hotspots, including platform-specific information dissemination, tailored educational interventions for diverse patient populations, efforts to enhance quality of life, and challenges related to health misinformation. Thematic evolution demonstrated a shift from basic information-seeking behaviors to broader topics such as digital health and health equity, indicating a multidimensional and interdisciplinary research trajectory.

**Conclusions:**

This study represents the first bibliometric analysis of social media–based cancer education, providing actionable insights to inform digital health literacy strategies and advance patient-centered, equitable health care.

## Introduction

The World Health Organization (WHO) projects that by 2040, the global incidence of new cancer cases will reach 28.4 million annually [1]. Despite the rising incidence of cancer, significant improvements in early screening, targeted therapies, and immunotherapies over the past 2 decades have notably enhanced the survival rates of patients with cancer. According to 2023 surveillance data, the overall 5-year cancer survival rate in the United States has increased from 49% in 1975 to 68%, while the rate for childhood cancers has surpassed 85% [2]. The cancer care paradigm is shifting from a disease-centered model to a health management–oriented model, with patient education emerging as a crucial element [3]. However, traditional offline medical education models face significant challenges regarding timeliness, accessibility, and personalization, creating barriers for health education systems [4].

Against this backdrop, the exponential growth of social media presents a transformative opportunity to overcome the limitations of traditional education models for patients with cancer. With over 5 billion global users, social media platforms have emerged as a novel and powerful medium for education for patients with cancer [5]. The intersection of social media and education for patients with cancer represents a rapidly evolving field. Traditionally, patient education has been defined as a planned, systematic, and continuous process through which health care professionals deliver information, skills training, and emotional support to patients and their families or caregivers, enabling them to manage their health conditions and engage in clinical decision-making [6]. In digital environments, however, patient education is often decentralized and informal, with content frequently emerging organically through information exchange among patients, caregivers, and peer communities, rather than being solely initiated by professionals [7]. Social media plays a significant role in patient empowerment, facilitating broader peer-to-peer education through the dissemination of health information [8]. Accordingly, this study expands the concept of education for patients with cancer to encompass any online content, produced by professionals or peers, that aims to enhance cancer-related knowledge, self-management capabilities, or decision-support. By leveraging real-time interactivity, personalized content delivery, and community-based support functions, social media–based cancer education not only provides timely and personalized information but also empowers patients by enhancing health literacy, encouraging active participation in decision-making, and fostering self-management awareness [9]. These elements collectively promote a dynamic balance of medical power between professional institutions and individual patients.

Social media are internet-based platforms that allow users to create and share content, as well as engage in social interactions [10]. They encompass various forms of media, including instant messaging tools, professional knowledge-sharing platforms, interactive platforms, video-sharing sites, and microblogging websites [11,12]. The proportion of patients with cancer obtaining disease-related information through social media has increased from 38% in 2015 to 72% in 2023 [13]. Moreover, the usage rate of social media among patients with cancer is 47% higher than that of the general population [14]. Through online interactive platforms, patients with cancer and their families can access a range of educational services, including the dissemination of disease-related knowledge, support for treatment decision-making, psychological and emotional support, and health behavior interventions [15]. This technology-enabled educational model provides personalized health information and promotes interaction and support among patient groups, transforming patients from mere recipients of treatment to active health managers.

However, the application of social media in education for patients with cancer faces significant risks in the information ecosystem. On major global platforms, such as YouTube, Facebook, and TikTok, a lot of cancer-related content lacks evidence-based support, potentially causing clinical harm [16]. For instance, 12% of posts related to bladder cancer on Pinterest advocate urine therapy [17]. Algorithm-driven recommendation mechanisms further exacerbate information asymmetry through the “echo chamber effect” [18]. Misinformation significantly influences patient decision-making. Commercialization further distorts medical education. Commercialization of health care significantly influences medical students’ ethical stance, with a high proportion agreeing to commercial propositions [19].

Currently, research on social media–based cancer education primarily focuses on enhancing information dissemination [20], technology-driven interventions [21], patient behavior analysis [22], and information quality assessment [23]. These studies are mostly empirical case analyses, emphasizing the application of social media in psychological support, treatment decision-making, and quality of life improvement. However, research in this field is increasingly fragmented, hindering a comprehensive understanding of the current status and developmental trajectory of social media–based education for patients with cancer. This fragmentation results in a lack of systematic insights into the knowledge structure, collaborative networks, and evolutionary pathways of the field. Although systematic reviews have investigated the application of mobile health interventions in education for patients with cancer [24] and the determinants of eHealth literacy among patients with cancer [25], they typically focus on specific interventions or influencing factors, overlooking the broader applications and research progress of social media in education for patients with cancer.

Bibliometric analysis provides a powerful framework for understanding the research landscape. Through a systematic study of academic literature, it clarifies the knowledge structure, emerging themes, collaborative networks, and developmental trajectories of a particular field, thereby offering evidence-based insights for researchers, policy makers, and platform developers. Previous studies have used bibliometrics to investigate the application of knowledge management on Twitter in health promotion [26], the interdisciplinary application of big data in HIV research [27], and the trends in natural language processing in the medical field [28]. However, to our knowledge, no bibliometric studies have specifically targeted social media–based education for patients with cancer.

This study used bibliometric methods to systematically analyze the current research landscape of education for patients with cancer on social media platforms and explore the knowledge structure and developmental trends in this field. By conducting quantitative analyses of publication trends, co-occurrence of high-frequency keywords, thematic clustering, core authors, and institutional collaborative networks, this study aimed to address the following research questions:

1. What are the core journals, institutions, and researchers in the field of social media–based education for patients with cancer?
2. How do different countries and regions collaborate in the field of social media–based education for patients with cancer?
3. What are the main themes and popular research directions in the field of social media–based education for patients with cancer?

By answering these questions, this study aimed to provide a theoretical integration pathway for academia, policy makers, and health care practitioners. It offers evidence-based insights for health policy makers to optimize the allocation of digital health education resources and data support for health care practitioners to design patient education programs tailored to the characteristics of the platform, thereby promoting patient empowerment.

## Methods

### Data Source and Retrieval Strategy

The Web of Science Core Collection (WoSCC) served as the data source for the present bibliometric and thematic analysis. This database covers peer-reviewed journals indexed in the Science Citation Index Expanded, Social Sciences Citation Index, and Emerging Sources Citation Index, ensuring broad coverage of high-quality interdisciplinary scholarly literature, and is widely used in bibliometric research [29,30].

To ensure a systematic and comprehensive retrieval process, and based on this study’s conceptualization of social media and patient education, we developed a search strategy integrating three key conceptual domains: (1) patients with cancer, (2) social media and digital platforms, and (3) patient education and health communication. The search terms in each domain were informed by Medical Subject Headings (MeSH) and frequently used synonyms identified in relevant literature. The retrieval process consisted of 5 steps.

#### Step 1: Identification of Articles Related to Patients With Cancer

In step 1, the following search query was used (search query #1): TS=(“cancer patients” OR “oncology patients” OR “neoplasm patients” OR “cancer survivors” OR “patients with cancer” OR “malignancy patients”). This ensured the inclusion of studies addressing patients with cancer or survivors described using various commonly adopted terms.

#### Step 2: Identification of Articles Related to Social Media and Online Communities

In step 2, the following search query was used (search query #2): TS=(“social media” OR “online platforms” OR “online communities” OR “Twitter” OR “Facebook” OR “Instagram” OR “YouTube” OR “TikTok” OR “LinkedIn” OR “Reddit” OR “WhatsApp” OR “WeChat” OR “Telegram” OR “blogs” OR “microblogs” OR “forums” OR “wikis”). This selection reflected both generic terms and platform-specific keywords, covering a range of functional types as well as regionally representative social media platforms to maximize the comprehensiveness of the search.

#### Step 3: Identification of Articles Related to Health Education and Communication

In step 3, the following search query was used (search query #3): TS=(“patient education” OR “health education” OR “health promotion” OR “health literacy” OR “health coaching” OR “health information” OR “patient engagement” OR “patient empowerment” OR “self-management education” OR “health communication” OR “patient support” OR “cancer education” OR “oncology education” OR “treatment adherence education” OR “symptom management education” OR “survivorship education”). This search covered a broad range of educational strategies and communication approaches relevant to cancer care and survivorship.

#### Step 4: Combining Queries and Applying Filters

We applied Boolean logic to combine the queries and retrieve articles situated at the intersection of these domains. The retrieval was conducted on March 5, 2025. The final search strategy was as follows: TS=(“cancer patients” OR “oncology patients” OR “neoplasm patients” OR “cancer survivors” OR “patients with cancer” OR “malignancy patients”) AND TS=(“social media” OR “online platforms” OR “online communities” OR “Twitter” OR “Facebook” OR “Instagram” OR “YouTube” OR “TikTok” OR “LinkedIn” OR “Reddit” OR “WhatsApp” OR “WeChat” OR “Telegram” OR “blogs” OR “microblogs” OR “forums” OR “wikis”) AND TS=(“patient education” OR “health education” OR “health promotion” OR “health literacy” OR “health coaching” OR “health information” OR “patient engagement” OR “patient empowerment” OR “self - management education” OR “health communication” OR “patient support” OR “cancer education” OR “oncology education” OR “treatment adherence education” OR “symptom management education” OR “survivorship education”).

#### Step 5: Refinement of the Results

To ensure information accuracy, a comprehensive data cleaning process was applied. Data were examined, with duplicates removed and erroneous entries corrected to ensure that each sample in the final dataset retained only 1 valid affiliation. To ensure rigor and consistency, the screening process was conducted by 2 independent researchers (ZG and GZ). Any discrepancies between the 2 researchers were resolved by consulting a third researcher (YF). Publications were limited to English-language articles within the thematic scope of this study, while meeting abstracts, editorial materials, proceeding papers, and studies unrelated to the topic were excluded. The literature screening was carried out using both the built-in refinement tools of the WoSCC database and manual review to ensure accuracy.

### Data Analysis and Visualization

This study used multisource analysis tools (VOSviewer 1.6.18, Bibliometrix R package 4.0, and CiteSpace 6.4.R2) to conduct multilevel analysis of the filtered literature dataset, covering 3 aspects: data standardization, collaboration network modeling, and research hotspot mining.

The original data exported from the WoSCC database included 2 types of data structures: one was in the plain text file format, which was used for analysis with VOSviewer and CiteSpace, and the other was in the BibTeX format, which was used for analysis with Bibliometrix. The record content of the exported data included “Full Record and Cited References,” which facilitated subsequent analysis.

During data standardization, bibliographic metadata were first standardized using regular expressions, and institution names were normalized via string-matching technology. For instance, different spelling variants of “University of Minnesota” were unified. In addition, different forms of the same keyword, such as “quality-of-life,” were merged into “quality of life” to ensure consistency. A keyword supplementation mechanism was established using the Python Pandas library: when author keywords were missing, they were supplemented with Keywords Plus data, and duplicate records were identified and removed through double verification of DOI and title.

Core journals were identified by Bradford Law in Bibliometrix. Journals were ranked in descending order according to the number of relevant articles published. Each zone contains approximately the same number of articles; however, the number of journals included in each successive zone increases geometrically. Among them, the first zone typically consists of a relatively small number of journals that publish the most relevant articles and is regarded as the core set of journals within the research field [31].

The collaboration networks of institutions and countries were generated using VOSviewer, with the normalization method set to LinLog/monotonic, a minimum collaboration frequency of 2, and a clustering resolution of 0.80. The size of the nodes represented the total link strength.

Research hotspot analysis included static and dynamic dimensions. In the static analysis, Bibliometrix generated a keyword tree map (top 50 high-frequency words; clustered by the Ward minimum variance method), theme map (top 200 high-frequency words; walktrap algorithm with step length t=4, minimum cluster frequency per thousand documents was set to 5), and factor analysis map (top 50 high-frequency words; optimal cluster number determined by the elbow method and a silhouette score ≥0.7). Semantic networks were constructed via VOSviewer, which created a keyword clustering map (binary counting; minimum co-occurrence frequency of 2, including 174 keywords). Co-occurrence intensity was calculated based on cosine similarity, and the minimum spanning tree algorithm formed the network topology. Clustering labels were automatically annotated using the association strength algorithm.

In the dynamic evolution analysis, CiteSpace version 6.4.R2 was used to construct a disciplinary category evolution network. The time span was set from 2011 to 2025, with a time slice length of 1 year. Node selection was based on the improved g-index, with the scaling factor k set to 25. The filtering criterion for keywords, terms, or stacked labels was “By Degree,” with the threshold set at 2. In the clustering analysis, the log-likelihood ratio algorithm was applied to identify disciplinary clusters, ensuring that the modularity Q value exceeded 0.3 to guarantee the rationality and clarity of the clusters. To preserve the integrity of the original network structure, no pruning operations were applied in the network trimming settings. Finally, the historical evolution trajectories of research topics were presented through the timeline view, revealing the dynamic evolutionary trends of disciplinary development.

### Ethical Considerations

This metadata analysis of published work used publicly available data and did not involve human subjects, individual data collection, biomedical interventions, or the use of any sensitive personal information. It is thus exempt from ethical review under national guidelines, and approval from an ethics committee was not required.

## Results

### Basic Information on the Included Studies

**Figure 1.**
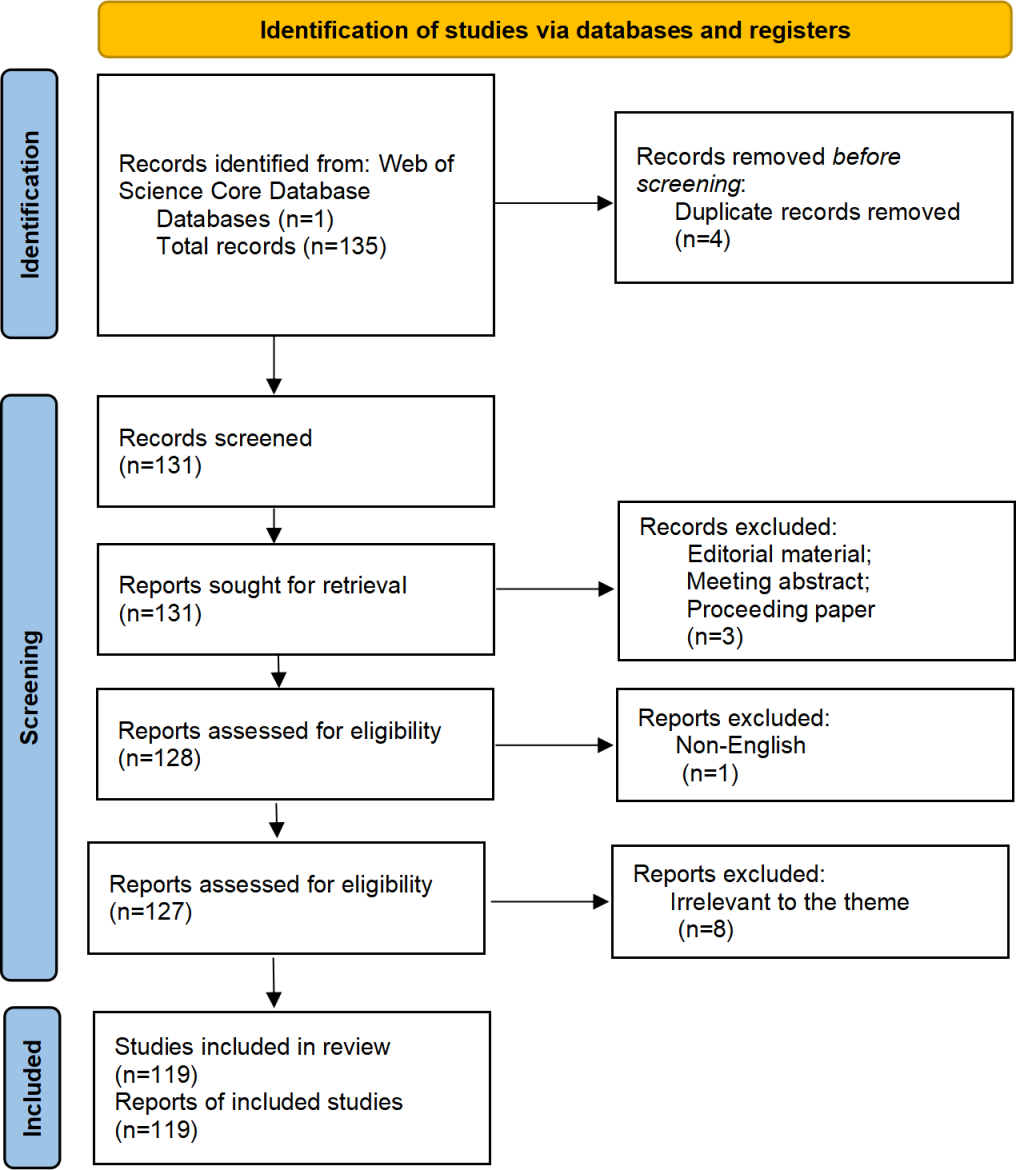
PRISMA (Preferred Reporting Items for Systematic Reviews and Meta-Analyses) flow diagram.

The relevant literature in this field covers the period from 2011 to 2025, involving 63 sources and producing a total of 119 publications, with an annual growth rate of 10.41%. The PRISMA (Preferred Reporting Items for Systematic Reviews and Meta-Analyses) flow diagram is presented in Figure 1. The research involved 784 authors, among whom only 2 were single authors, and 17.65% of the authors were involved in international collaborations. The average number of coauthors per publication was 7. The research included 414 author-provided keywords, with a total of 4953 references cited across the publications. The average age of the publications was 4.52 years, and each publication was cited on average 24.39 times, reflecting the continuity and academic impact of research in this field (Multimedia Appendix 1).

**Figure 2.**
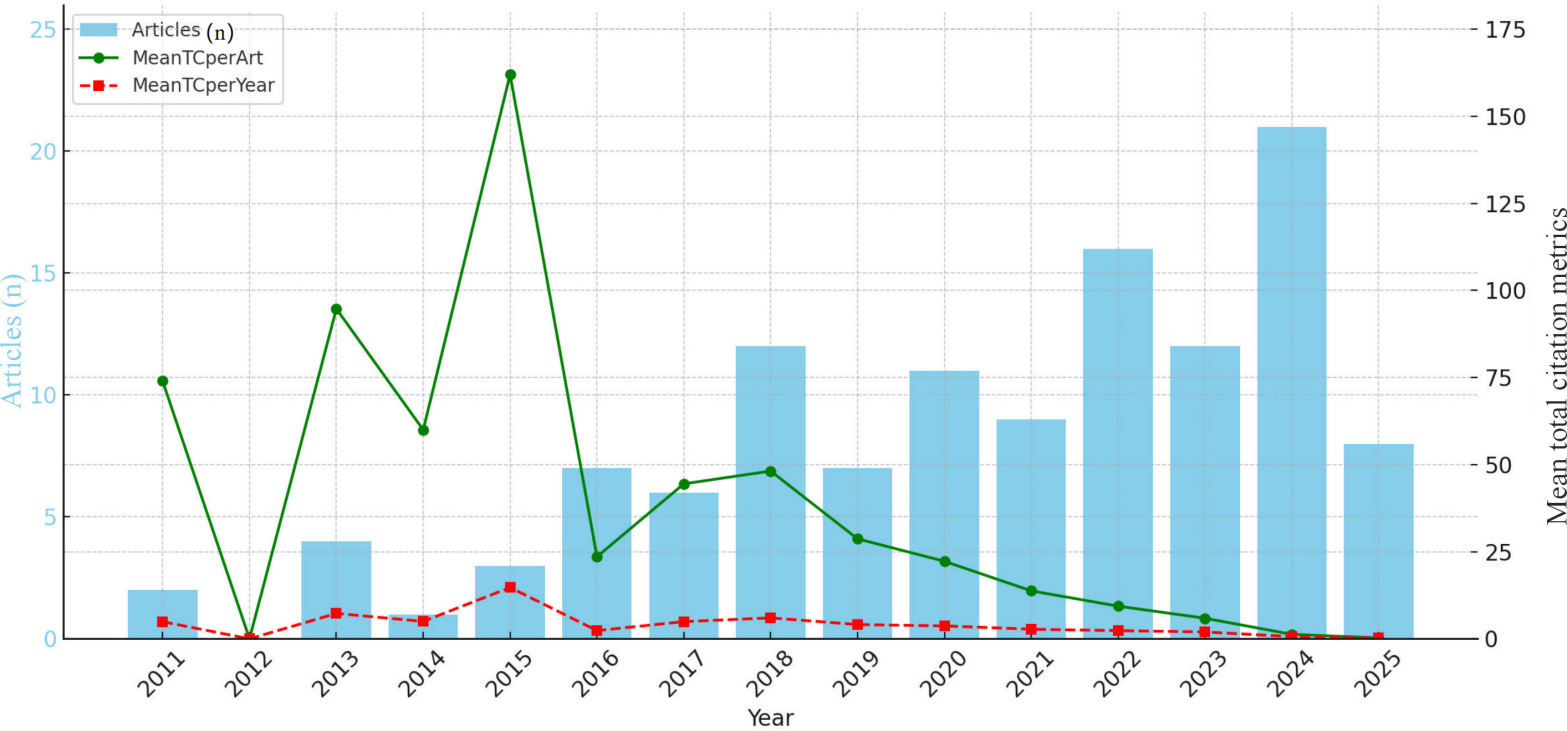
Evolution chart of article output and mean total citation metrics (2011-2025). MeanTCperArt: mean total citations per article; MeanTCperYear: mean total citations per year.

Figure 2 presents the evolution chart of article output and mean total citation metrics. The annual publication output fluctuated but showed an overall upward trend, with slight declines in 2014, 2017, 2019, and 2021. Regarding mean total citation metrics, both mean total citations per article and mean total citations per year displayed a general downward trend.

### Analysis of the Most Productive Entities

Table 1 presents the high-output journals, institutions, and authors in the study field. Regarding journals, the Journal of Medical Internet Research led with 13 publications (10.9%), followed by Supportive Care in Cancer with 10 publications (8.4%). The Journal of Cancer Education and the Journal of Cancer Survivorship each had 8 publications (6.7%).

**Table 1.** The top 10 journals, institutions, and authors by publication output.

Variable	Articles, n
Journal	
Journal of Medical Internet Research	13
Supportive Care in Cancer	10
Journal of Cancer Education	8
Journal of Cancer Survivorship	8
BMJ Open	4
Cancer Medicine	3
International Journal of Medical Informatics	3
Journal of Adolescent and Young Adult Oncology	3
Psycho-Oncology	3
BMC Cancer	2
Affiliation	
University of Minnesota	6
University of British Columbia	3
University Health Network	3
The Ohio State University	2
University College Cork	2
University of Colorado	2
University of Southern California	2
Bellvitge Biomedical Research Institute	2
University of Florida	2
Princess Margaret Cancer Centre	2
Author	
Aggarwal R	2
Balaratnam K	2
Beaupin LK	2
Bender J	2
Bender JL	2
Blanco-Diaz M	2
Brown MC	2
Chen J	2
Chou WYS	2
Davoren	2

In terms of institutions, the University of Minnesota had the highest number of publications (6/119, 5.0%). Both the University of British Columbia and the University Health Network were tied for second place, with 3 publications each (2.5%). The majority of these high-output institutions are located in the United States (5 institutions) and Canada (3 institutions), with 1 additional institution in Ireland (University College Cork) and 1 in Spain (Bellvitge Biomedical Research Institute).

In terms of authors, no single author stood out with a significantly higher number of publications. Most authors in the list had published 2 articles (eg, Aggarwal R and Balaratnam K). This indicated a relatively dispersed research community in this field, without a clear core group of high-output authors.

Using Bibliometrix, we identified 4 core journals in the field of social media–based education for patients with cancer. These include the Journal of Medical Internet Research, Supportive Care in Cancer, Journal of Cancer Education, Journal of Cancer Survivorship, and BMJ Open (Multimedia Appendix 2).

**Figure 3.**
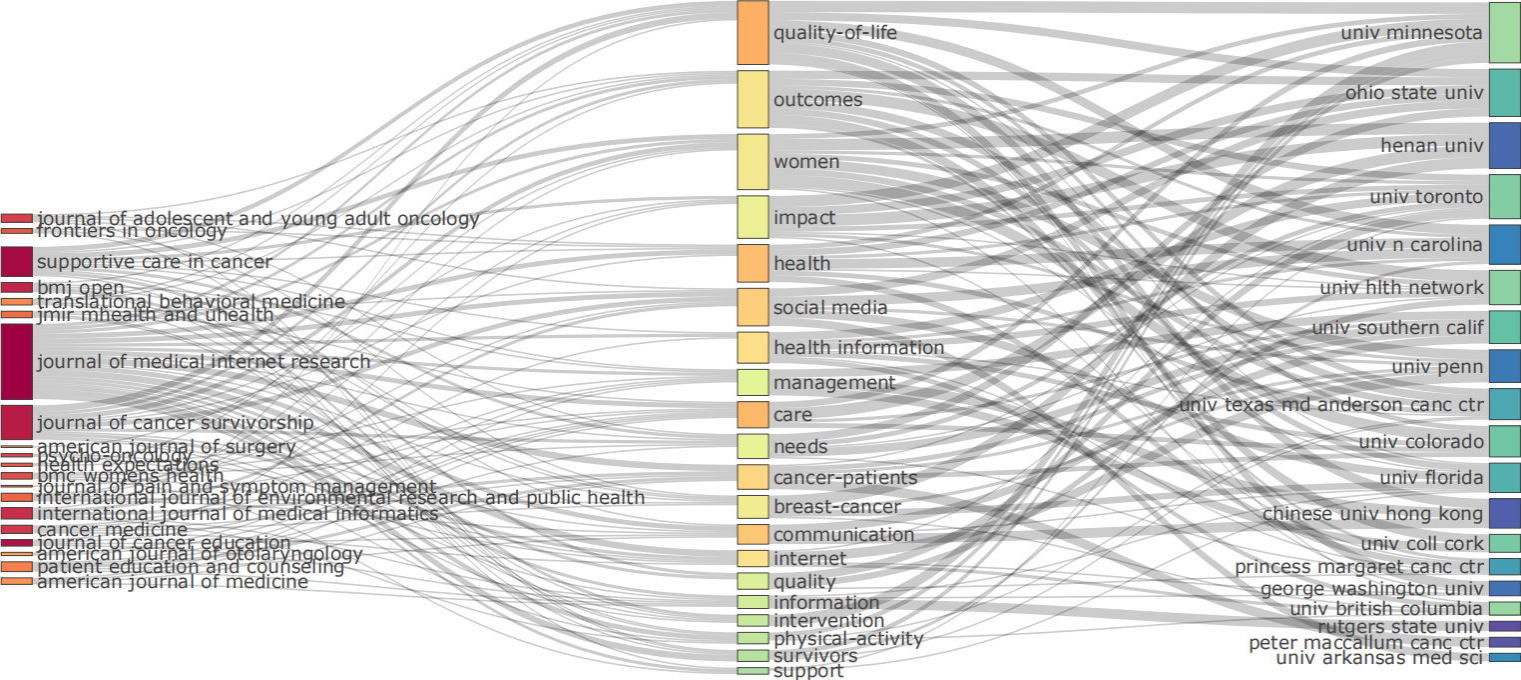
Sankey diagram of relationships among journals (left), keywords (center), and institutions (right).

The Sankey diagram (Figure 3) generated by Bibliometrix provides a visual representation of the interconnections among journals, keywords, and institutions, thereby elucidating the research themes of the most influential journals and institutions in the field of social media–based education for patients with cancer.

The Journal of Medical Internet Research, the most productive journal in this field, is distinguished by its extensive range of topics. The keywords associated with it included “social media,” “cancer patients,” “health information management,” “communication,” “intervention,” and “quality of life.” These connections underscore its emphasis on the application of social media in education for patients with cancer, with a particular focus on core topics such as patient health information management, communication-based interventions, support system development, and the enhancement of quality of life through social media platforms. The University of Minnesota, a high-output institution in this domain, was linked to keywords such as “quality of life,” “outcomes,” “women,” and “impact.”

### Analysis of the Collaboration Network

With regard to the top 10 countries contributing to the field, based on the number of publications among the 119 articles analyzed, the United States had the highest number of publications (56/119, 47.1%), followed by Canada (12/119, 10.1%) and China (10/119, 8.4%). Other contributing countries included Australia and Spain (each 7/119, 5.9%); the United Kingdom and the Netherlands (each 5/119, 4.2%); and Turkey, Japan, and South Korea (each 3/119, 2.5%) (Multimedia Appendix 3).

**Figure 4.**
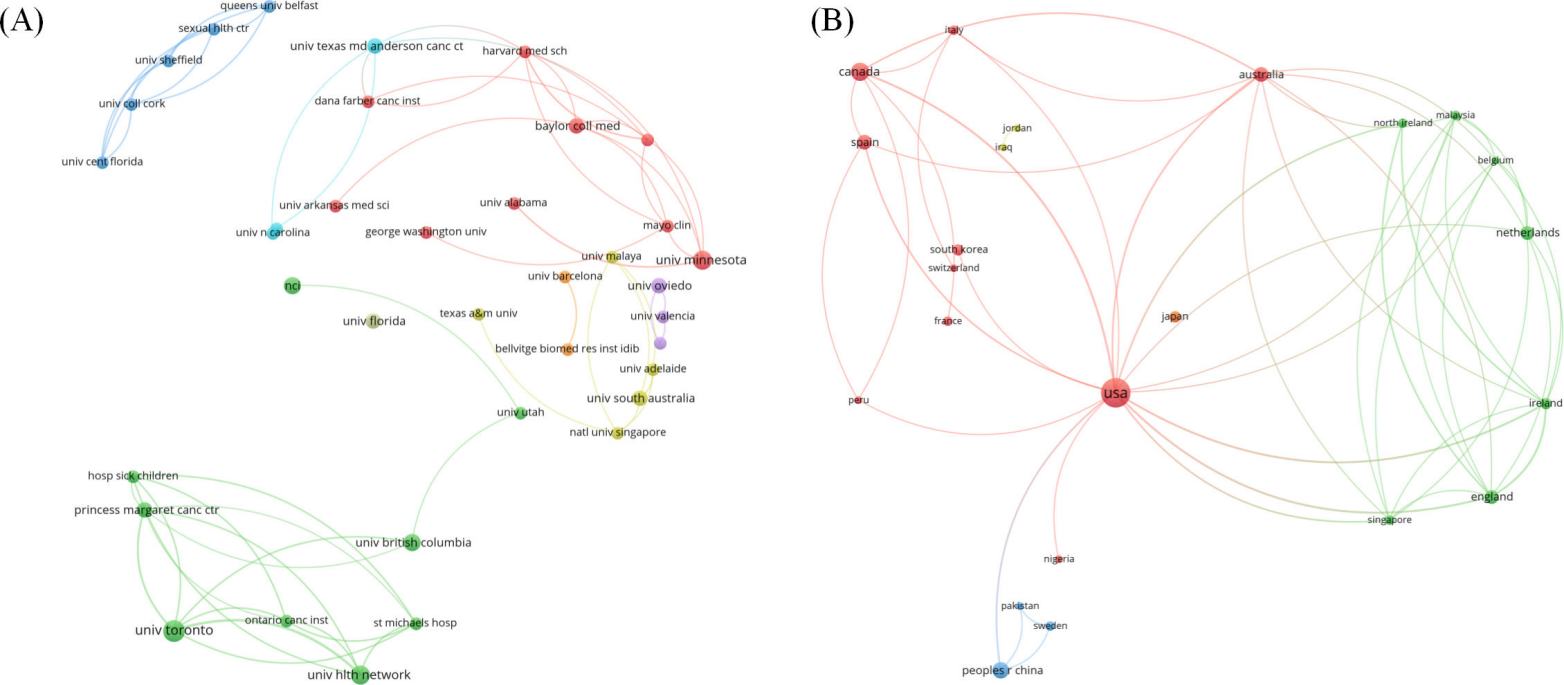
Collaboration analysis. (A) Institution collaboration. The green cluster is centered around the University of Toronto, and the blue cluster is primarily composed of institutions from Northern Ireland such as Queen's University Belfast and The University of Sheffield. The red cluster includes prominent American institutions like Harvard Medical School and Baylor College of Medicine, while the purple cluster is dominated by Spanish institutions. The colored lines between nodes reveal regional cooperation patterns, with significant collaboration among institutions in the southern United States. (B) Country collaboration. The United States occupies a central and pivotal position in international cooperation.

The collaboration analysis diagrams were generated by VOSviewer. The institution collaboration diagram (Figure 4A) illustrates the cooperative relationships among various institutions, with node size indicating their importance within the network. Overall, the collaboration network exhibited distinct regional characteristics and was primarily led by institutions from North America and Europe.

The national collaboration diagram (Figure 4B) illustrates the cooperative relationships among various countries. The United States was interconnected with multiple countries, including Canada, the Netherlands, and Ireland, through dense collaborative links, thereby steering the core dynamics of international cooperation. Concurrently, countries, such as Spain, Italy, Australia, and Belgium, also exhibited extensive international collaborative networks.

### Analysis of Research Hotspots

**Figure 5.**
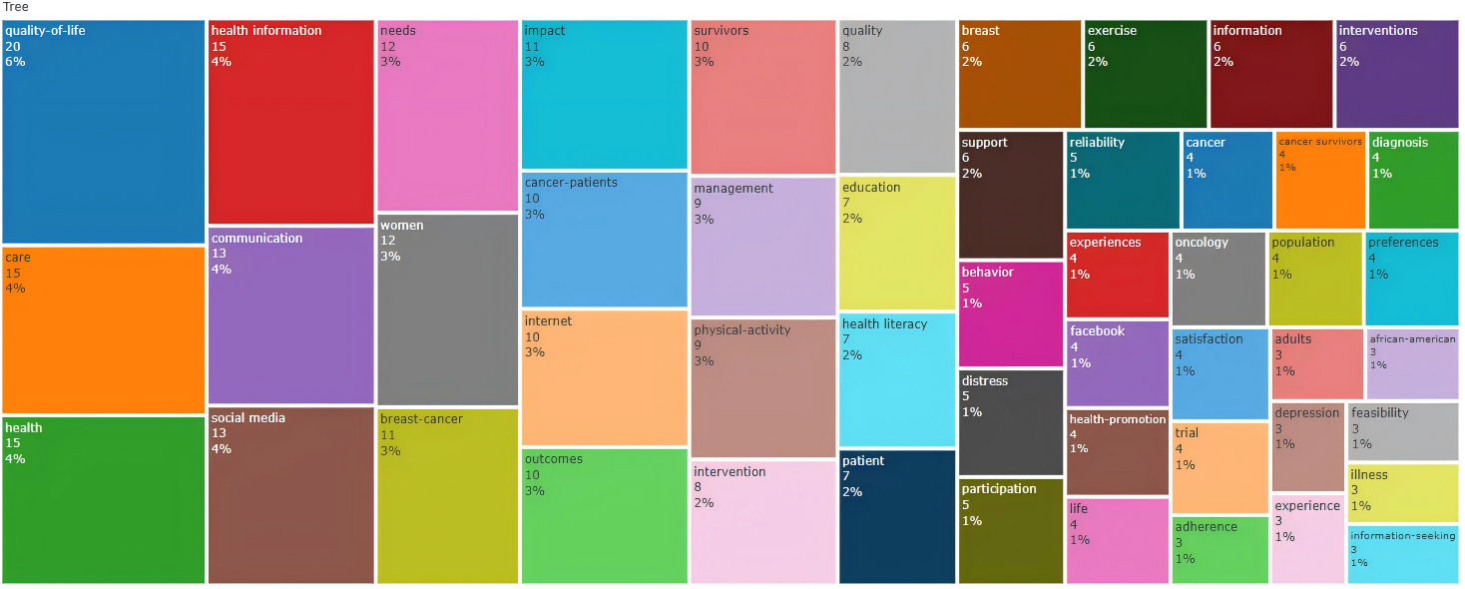
Keyword tree map of social media–based cancer education.

The keyword tree map generated by Bibliometrix (Figure 5) identifies the 50 most frequent keywords in social media–based education for patients with cancer. Core keywords, such as “quality of life” (20/354, 6%), “care” (15/354, 4%), and “health” (15/354, 4%), were prominent, reflecting a focus on patient well-being. Other high-frequency keywords were related to demographics (eg, “women” [12/354, 3%], “cancer survivors” [4/354, 1%], and “African-American” [3/354, 1%]), health communication (eg, “health information” [15/354, 4%], “communication” [13/354, 4%], and “education” [7/354, 2%]), behavioral support (eg, “exercise” [6/354, 2%], “behavior” [5/354, 1%], and “physical activity” [9/354, 3%]), and psychological metrics (eg, “distress” [5/354, 1%] and “reliability” [5/354, 1%]). These keywords underscore the multidimensional nature of research in this area.

**Figure 6.**
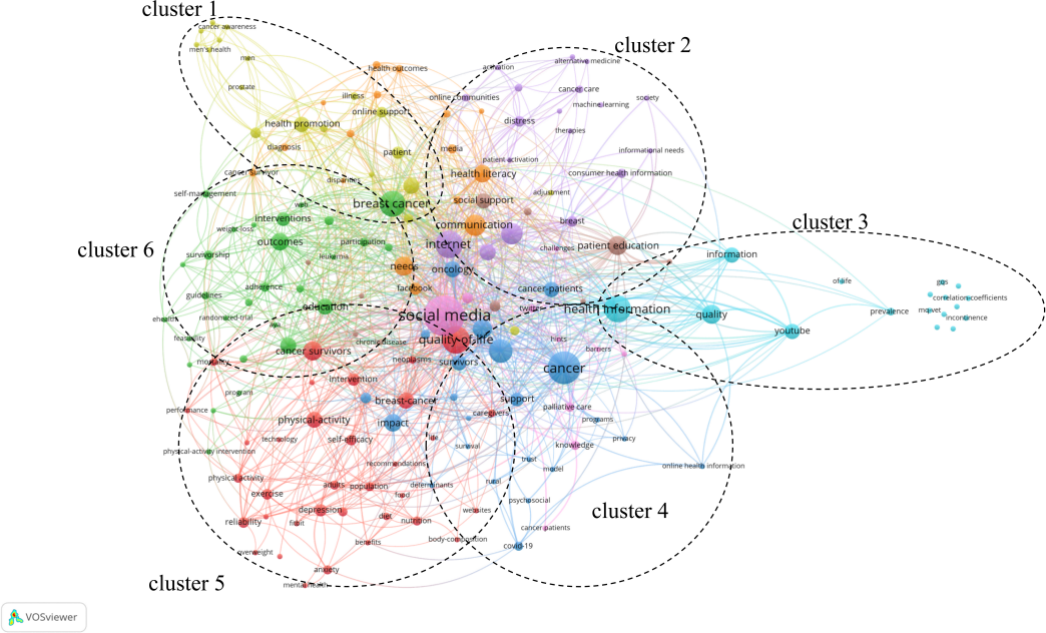
Keyword clustering of social media–based cancer education.

Based on the keyword tree map, a further keyword clustering analysis (Figure 6) was conducted to reveal the strength of co-occurrence relationships among keywords within the literature, generated using VOSviewer. A total of 174 keywords were included. The clustering map categorized the keywords into 6 distinct clusters, each coalescing around a specific thematic focus.

Cluster 1 centered on cancer-related health promotion and men’s health issues. Its keywords were “health promotion,” “men,” “prostate,” “cancer awareness,” and “diagnosis.” The cluster explored cancer prevention strategies and the diagnosis of prostate diseases in men.

Cluster 2 focused on patient experience and health disparities, particularly the role of complementary and alternative medicine in cancer care. Keywords, such as “patient,” “health outcomes,” “disparities,” “alternative medicine,” and “complementary,” suggested that this cluster centers on understanding variations in health outcomes among different patient populations. Particular emphasis was placed on the influence of cultural and social contexts on treatment effectiveness and the integration of traditional and alternative medicine approaches to improve therapeutic outcomes.

Cluster 3 dealt with cancer information dissemination and online resource use. Its keywords were “information,” “internet,” “social media,” “health information,” and “YouTube.” It discussed how cancer information spreads via the internet and social media, and how patients access health resources online.

Cluster 4 covered care for patients with cancer and relevant medical technologies. Its keywords were “cancer,” “cancer patients,” “palliative care,” “machine learning,” and “surgery.” The cluster focused on care for patients with cancer, especially palliative care, and the application of medical technologies like machine learning in cancer treatment, including surgical assistance.

Cluster 5 looked into cancer survivor well-being and lifestyle interventions. Its keywords were “cancer survivors,” “quality of life,” “physical activity,” “mental health,” and “nutrition.” The cluster assessed how cancer survivors can enhance their quality of life through physical activity and nutrition, and emphasized their mental health care.

Cluster 6 focused on breast cancer research and the support needs of patients throughout the treatment process. Keywords, such as “breast cancer,” “communication,” “support,” “outcomes,” and “randomized controlled trial,” highlighted a dual emphasis: the use of rigorous methodologies, particularly randomized controlled trials, to evaluate treatment efficacy, and the importance of ensuring comprehensive communication and emotional support for patients during the course of care.

**Figure 7.**
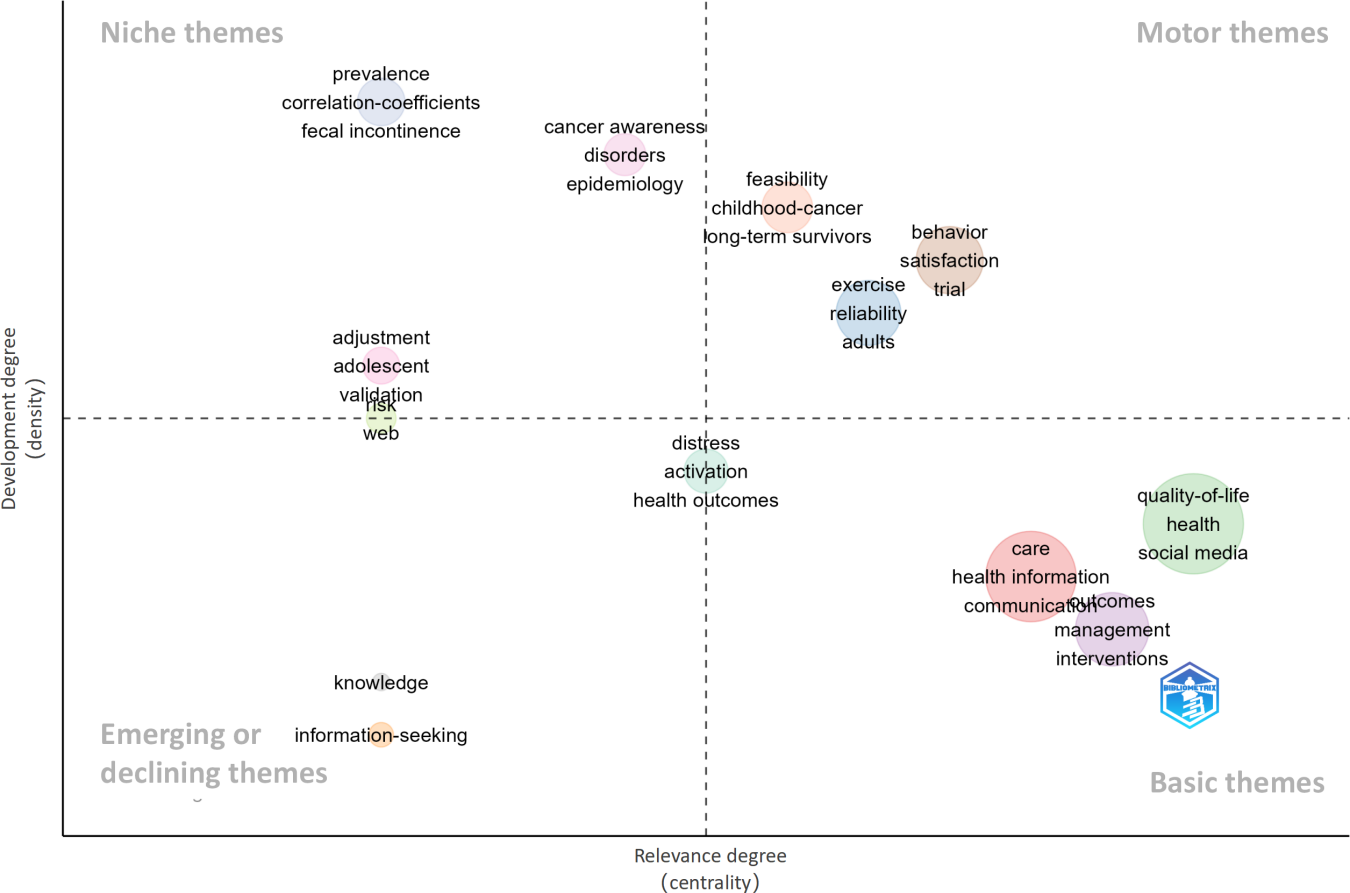
Thematic map of social media–based cancer education. The horizontal axis represents the centrality of a theme in the field (ie, its relevance), while the vertical axis represents the density (ie, the degree of development of the theme). The division into 4 quadrants helps to interpret the position and evolutionary direction of each theme within the field.

In addition to the keyword clustering analysis, a thematic map (Figure 7) was generated using Bibliometrix to further reveal the structural distribution and development potential of research themes in cancer education on social media platforms. The map had 4 quadrants.

The upper-right quadrant had “motor themes” with key themes that were both highly relevant and well developed. Keywords, such as “feasibility,” “childhood cancer,” “long-term survivors,” “behavior,” “satisfaction,” “trial,” and “adults,” indicated that current research is focused on the feasibility of clinical interventions, the behavioral management of cancer survivors, and the validation of educational pathways grounded in behavioral theories.

The upper-left quadrant had keywords, such as “prevalence,” “epidemiology,” “correlation coefficients,” “cancer awareness,” and “adolescent,” which pointed to research centered on specific populations, disease types, or methodological approaches. Although these studies are relatively mature in terms of methods and theoretical frameworks, they have yet to exert a broad influence in the field due to limitations in target populations or research scope.

The lower-right quadrant included keywords, such as “quality of life,” “health,” “social media,” “care,” “communication,” and “interventions.” These represented foundational topics that recur within cancer education, covering aspects of health information dissemination, care management, and quality of life interventions. While these themes are highly relevant to the field, their internal mechanisms still require further theoretical development and empirical exploration, and they remain at a critical stage of transitioning from empirical observations to theoretical frameworks.

The lower-left quadrant had keywords, such as “knowledge” and “information-seeking,” which were classified as either emerging or declining themes, indicating that they currently lack methodological support or practical integration.

In addition, keywords, such as “distress” and “activation,” were positioned near the boundary of the left quadrants, while “risk” and “web” appeared between the upper-left and lower-left quadrants. These “boundary themes” suggest possible areas for interdisciplinary convergence and research integration.

**Figure 8.**
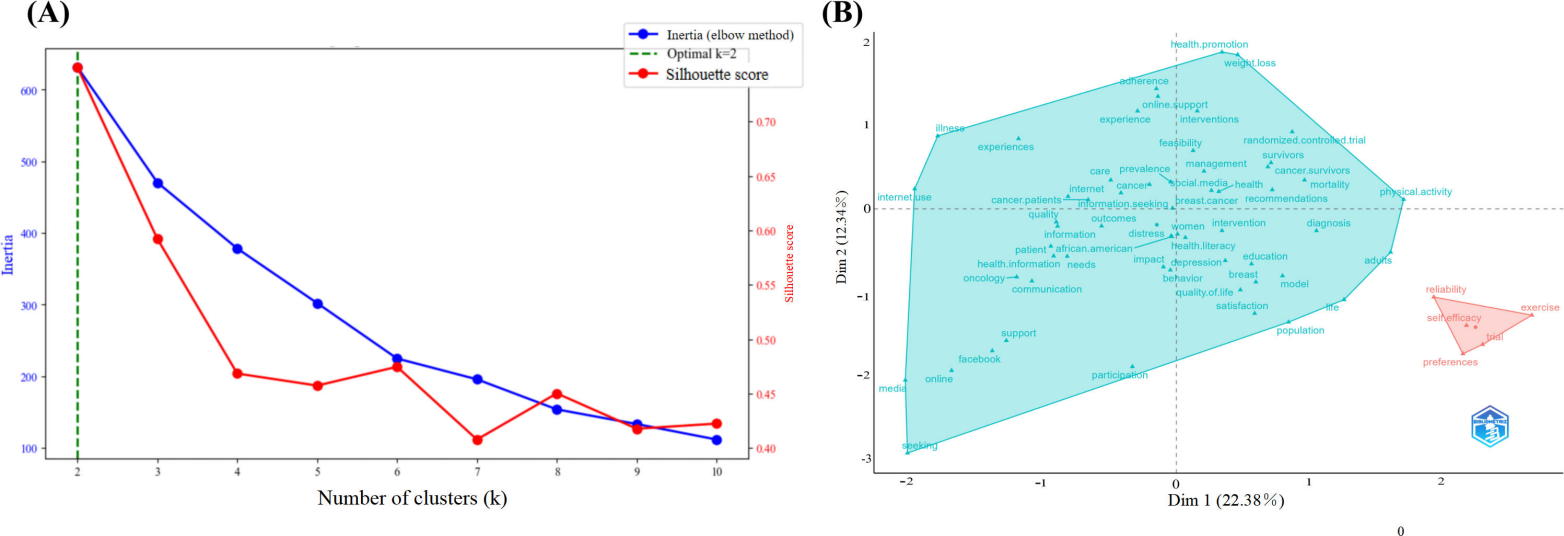
Factor analysis of social media–based cancer education. (A) Determination of the optimal number of clusters; (B) Structure map.

The factor analysis conducted using Bibliometrix further examined the covariation among keywords from the perspective of latent variables, contributing to the extraction of the main research pathways and logical structure [32]. The optimal number of clusters was identified as 2 (Figure 8A). Figure 8B illustrates the specifics of the clustering with red and blue clusters.

The red cluster included keywords, such as “reliability,” “self-efficacy,” “trial,” “exercise,” and “preferences,” emphasizing the validation of short-term interventions through standardized experiments. This represented an individualized intervention pathway guided by medical empiricism.

The blue cluster covered a broader scope, including keywords related to social media and information access, such as “internet use,” “social media,” “facebook,” “information seeking,” and “health information needs.” It also covered keywords related to disease management and quality of life, such as “illness,” “experiences,” “behavior,” “quality of life,” “health promotion,” and “weight loss;” keywords associated with support and interventions, such as “online support,” “interventions,” “education,” and “management;” and keywords related to implementation and management, such as “feasibility,” “management,” “randomized controlled trial,” “diagnosis,” and “physical activity.”

The blue cluster highlighted patient-centered psychosocial support and cultural adaptability, whereas the red cluster focused more on the evidence-based validation of individual behavioral interventions. Together, these clusters represented two distinct directions: (1) the empirical validation pathway of individualized behavioral interventions and (2) the systemic construction of a population health ecosystem.

**Figure 9.**
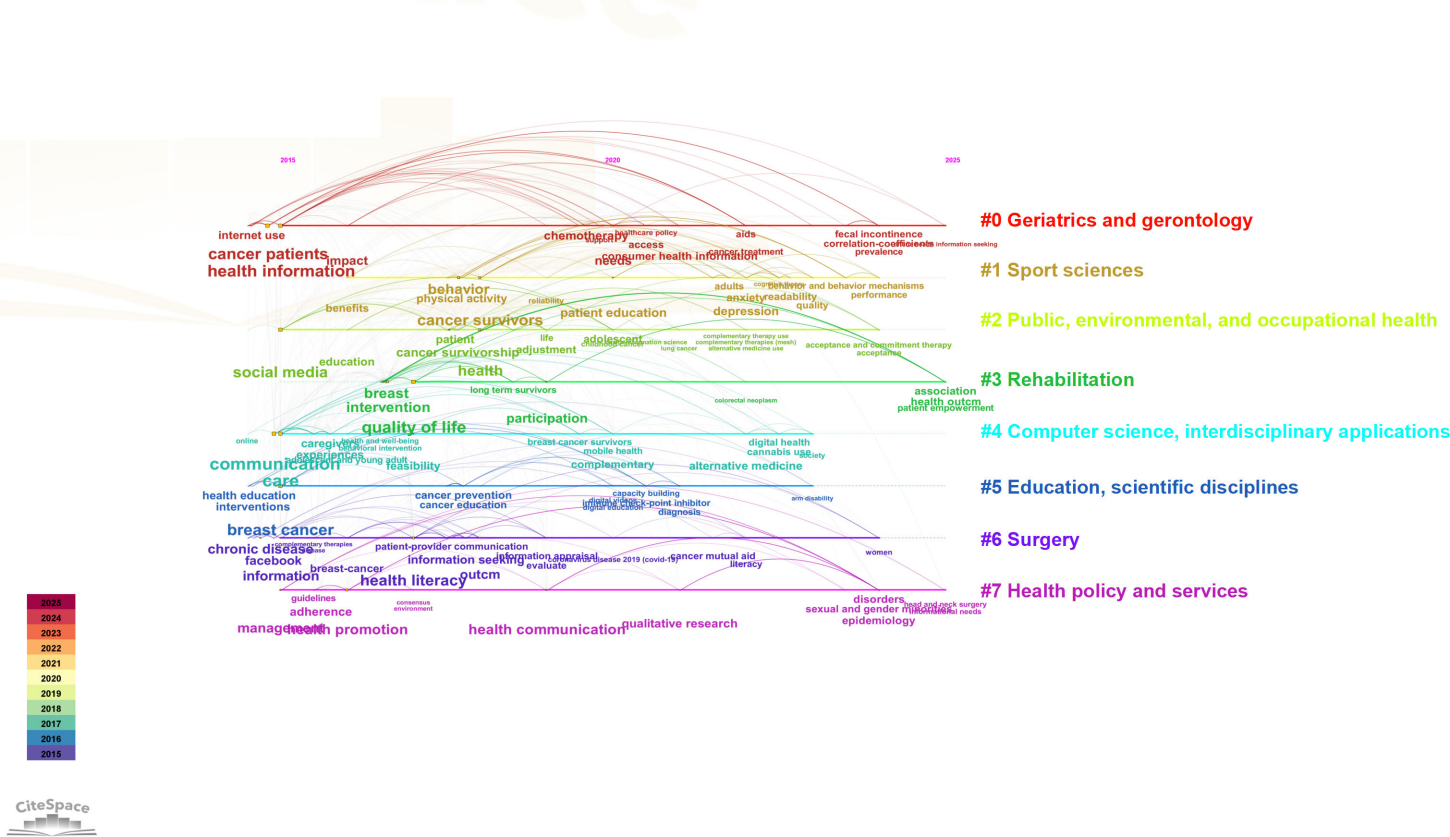
Evolutionary map of keyword disciplinary categories of social media–based cancer education. On the right, 8 disciplinary categories are labeled, while on the left, keywords are color-coded along a timeline, indicating their level of activity during different time periods.

Figure 9 presents the evolutionary map of keyword disciplinary categories generated by CiteSpace, illustrating the disciplinary domains and temporal distribution of keywords in cancer education research based on social media platforms. The figure demonstrates the evolving trends of keywords related to cancer across various disciplinary fields. Overall, research in cancer education and information dissemination is undergoing a multidimensional transformation, from an early-stage focus on information access and patient education to more integrated concerns, including comprehensive treatment, psychosocial support, digital empowerment, and health equity.

In the early stage, multiple disciplines primarily focused on keywords, such as “health information,” “patient education,” “internet use,” and “communication,” reflecting a strong emphasis on basic information acquisition and health education for patients with cancer. This trend was particularly evident in fields such as “geriatrics and gerontology,” “sport sciences,” and “education, scientific disciplines.”

In the middle stage, research across disciplines began to concentrate on specific treatments and health interventions, as indicated by the frequent appearance of keywords such as “chemotherapy,” “cancer treatment,” “intervention,” and “health care policy.” This shift demonstrates a progression from information dissemination toward clinical pathways, rehabilitation strategies, and health policy development, with particular attention observed in the fields of “public, environmental, and occupational health,” “rehabilitation,” and “surgery,” highlighting concerns regarding treatment accessibility and policy support.

In the later stage, research trends became increasingly diversified and interdisciplinary. On one hand, keywords, such as “digital health” and “capacity building,” illustrated the growing role of “computer science, interdisciplinary applications” and “education, scientific disciplines” in integrating digital technologies with educational platforms. On the other hand, the emergence of keywords, such as “prevalence,” “patient empowerment,” “alternative medicine,” and “sexual and gender minorities,” suggested that cancer education research on social media is expanding toward topics related to individual differences, psychological cognition, and social equity. In particular, within the field of “health policy and services,” increasing attention is being directed toward psychological support and addressing the health needs of vulnerable populations.

In summary, the keyword evolution in cancer education research on social media platforms revealed distinct developmental phases—beginning with information acquisition and patient education, progressing through treatment modalities and behavioral interventions, and evolving into digital support systems and the social governance of health equity. This interdisciplinary and multidimensional evolution reflects not only the advancement of medical technologies but also the growing societal, cultural, and policy-driven demand for comprehensive cancer education and patient support.

## Discussion

### Basic Information

This study involved the first bibliometric analysis of research on social media–based cancer education, offering a comprehensive overview of the evolution, research distribution, and collaboration patterns in this field. The overall publication volume in this area has shown an upward trend; however, the mean total citations per article has declined each year, reflecting the foundational role of early basic research. The fluctuation in research output may be associated with the iterative development of social media platforms and technologies, as well as the impact of major social events such as the COVID-19 pandemic. Among journals, the Journal of Medical Internet Research has the highest number of publications (Table 1). It is distinguished by its disciplinary foresight, methodological innovation, and international influence, with a particular focus on core topics such as patient health information management, communication-based interventions, support system development, and the enhancement of quality of life through social media platforms. At the national level, the United States holds a dominant position in this field, which may be related to its state support [33]. Canada also demonstrates significant international influence in this field. Its government has promoted digital health innovation initiatives [34], and its research institutions have formed a clustered research ecosystem, particularly in North America and Europe [35]. Although China ranks third globally in publication volume, it lacks high-output institutions with international influence, reflecting issues of research fragmentation and insufficient internationalization (Figure 4). Other developing countries have relatively low research output in this field. Future efforts should prioritize stronger global resource-sharing and deeper international collaboration.

### Research Focuses and Implications

Through a systematic keyword analysis, we identified the most prevalent research themes in social media–based cancer education: platform-specific information dissemination, tailored educational interventions for diverse patient populations, efforts to enhance quality of life, and challenges related to health misinformation. The prominence of these themes indicates that social media plays a multifaceted role in education for patients with cancer.

We identified YouTube, Twitter, and Facebook as the most commonly used platforms for cancer education at present. Cancer education strategies should be tailored to the specific characteristics of each platform. On YouTube, a long-form video platform, health educators can leverage its rich audiovisual capabilities to develop structured learning modules, enabling patients to systematically acquire knowledge about treatment processes and side-effect management [36]. The real-time nature and high interactivity of Twitter make it particularly suitable for the rapid dissemination of evidence-based updates and expert interpretations, thereby facilitating the immediate spread of research findings and clinical guidelines [37]. Facebook’s group and mixed-content format is more conducive to building patient communities where experience sharing and emotional support can help enhance treatment adherence and psychological well-being [38]. Furthermore, since Twitter’s rebranding to X, the platform’s recommendation algorithm has undergone significant adjustments, which may alter its user demographics [39-41]. Future educational strategies must adapt to these changes by integrating strategic content planning and keyword optimization to improve information visibility and intervention effectiveness [42,43].

Keyword analysis highlighted the diversity of patient populations. Male patients tend to seek health support from official sources and are less likely to engage in emotional expression [44,45], and expert-delivered authoritative educational videos and professional consultations can be leveraged to enhance educational effectiveness. In contrast, female patients are more willing to share personal treatment experiences and emotions [46]; therefore, leveraging peer-to-peer exchange and emotional resonance can enhance the effectiveness of educational interventions [47]. Adolescents and young adults tend to prefer interactive and visual forms of education and are more susceptible to the influence of digital opinion leaders [48]. Therefore, short-form video content and dissemination mediated by digital opinion leaders should be used to enhance their engagement, complemented by strengthened online community building [49,50]. Elderly patients typically rely on traditional or official channels for health information and are more receptive to live lectures and voice-based questions and answers [51]. Thus, content should focus on simplicity, clarity, and strong practical applicability, with increased opportunities for face-to-face or synchronous communication. Vulnerable groups, such as African Americans and gender minorities, face barriers in health information access due to trust issues and cultural sensitivity [52,53]. Therefore, educational content should prioritize privacy protection and cultural adaptation [54-56]. Additionally, improving digital literacy among disadvantaged populations, including low-income individuals, rural residents, and the elderly, can help bridge the digital divide [57]. Targeted training, simplifying the complexity of digital applications, and increasing community support [58,59] can effectively close this gap.

Quality of life–focused interventions represent the core of social media–based cancer education. Studies have shown that such interventions can improve patients’ treatment adherence, self-management abilities, and psychological well-being [60]. Tools, such as 3D medical animations [61], short-form videos [62], and online courses [63], deliver information on postoperative care, rehabilitation training, psychological adjustment, and nutrition management in visual formats, thereby enhancing patients’ comprehension and retention of professional knowledge [64]. Artificial intelligence and big data–driven intelligent recommendation systems further provide personalized guidance on diet, exercise, and psychological support [65], while online consultations offer timely resources to alleviate anxiety and improve intervention outcomes [66]. When designing health content for diverse populations, it is essential to incorporate outcome-oriented feedback mechanisms. These can be implemented through regular surveys [67], online assessments [68], and user-generated satisfaction ratings [69]. Importantly, future research should integrate the authentic voices of patients with cancer as end-users of educational platforms by employing qualitative methods, such as in-depth interviews [70] and focus group discussions [71], to gather their experiences, needs, and suggestions. This approach not only enriches the dimensionality of feedback data, providing deeper and more contextualized insights, but also helps align educational content and delivery formats with patients’ actual needs and preferences [72]. Based on these quantitative and qualitative feedback mechanisms, health education platform managers should lead real-time adjustments to educational content and formats, with deep involvement from educational experts and health care professionals. Educational experts are responsible for optimizing content structure and pedagogical methods from an instructional perspective, while health care professionals ensure the scientific validity and accuracy of the content. Through such collaborative efforts, all stakeholders can ensure that educational content and formats are precisely tailored to patient needs, thereby maximizing educational effectiveness and improving patients’ quality of life, treatment adherence, and mental health.

It is important to note that the proliferation of false cancer information has become a significant challenge. Unverified alternative therapies, pseudoscientific advice, and distorted medical data (often emotionally provocative) tend to spread more rapidly and widely than evidence-based information [73,74]. Recommendation algorithms exacerbate this issue [75]. Studies indicate that approximately 30% of cancer-related information contains false or misleading elements [76], and patients with lower educational attainment are more susceptible to these effects [77]. The dissemination of misleading information not only delays treatment and undermines trust between patients and health care providers, but also increases patients’ psychological burden, making clinical decision-making more complex and uncertain [78]. Addressing this challenge requires multistakeholder collaboration, including enhanced media literacy education [79], encouraged participation of health care professionals in health communication [80], application of natural language processing and machine learning–based intelligent detection tools to identify and flag misleading content in real-time [81,82], and policy-level strengthening of content regulation to effectively curb the spread of misinformation.

### Limitations

This study has several limitations. First, relying solely on the WoSCC database may have resulted in the omission of relevant literature from other significant databases, undermining the comprehensiveness of the analysis. Additionally, focusing only on English-language publications overlooked research in other languages, potentially excluding region- or language-specific studies. Second, despite diligent keyword selection, some articles related to potential themes might have been overlooked, limiting the completeness of the analysis. Moreover, the selection of node parameters is relatively subjective. Different researchers may choose varying parameters based on research objectives and interpretations, which can introduce bias into the analysis results. Future research should address these issues by incorporating additional databases, including multilingual literature; integrating qualitative and quantitative approaches; and updating the data range, thereby enhancing the breadth and accuracy of the findings. Additionally, employing multiple parameter validation methods can improve the reliability and comparability of research results.

### Conclusions

This bibliometric analysis of 119 articles in the field of social media–based cancer education revealed trends in research output, characteristics of international collaboration, and key research hotspots. The Journal of Medical Internet Research emerged as the leading journal in this field, publishing the highest number of relevant articles. The University of Minnesota was the most productive institution, and the United States was the most productive country, with 5 of the top 10 institutions being based in the United States and international collaborations being led by the United States. Through keyword analysis, this study further identified research development phases and key research focuses in this field. This bibliometric analysis provides direction for future research, helping scholars better understand the evolution and development trends in this area. Future studies should further explore the mechanisms of social media platforms in education for patients with cancer, investigate innovative educational models and technological applications, and better address the diverse needs of patients with cancer to improve their quality of life and health literacy.

## Supplementary material

10.2196/77214 Multimedia Appendix 1 Basic information on the included studies involving social media–based cancer education research.

10.2196/77214 Multimedia Appendix 2 Core journals in social media–based cancer education research by Bradford Law.

10.2196/77214 Multimedia Appendix 3 Top 10 most productive countries and publication output of social media–based cancer education research.

## Abbreviations

WoSCC: Web of Science Core Collection
