# Supplementary figures and images for: Social Media–Based Cancer Education: Bibliometric and Thematic Analysis
Source: JMIR Cancer. 2025 Oct 6;11:e77214. doi: 10.2196/77214 (PMC12500228; doi:10.2196/77214)

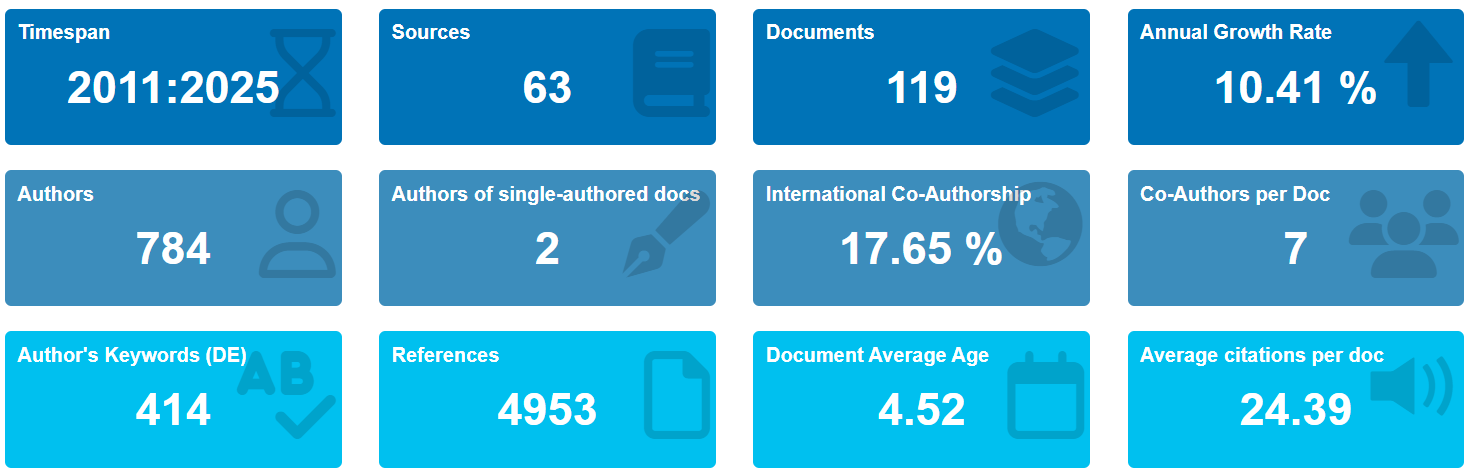

Supplement: Multimedia Appendix 1 [file cancer-v11-e77214-s001.png]

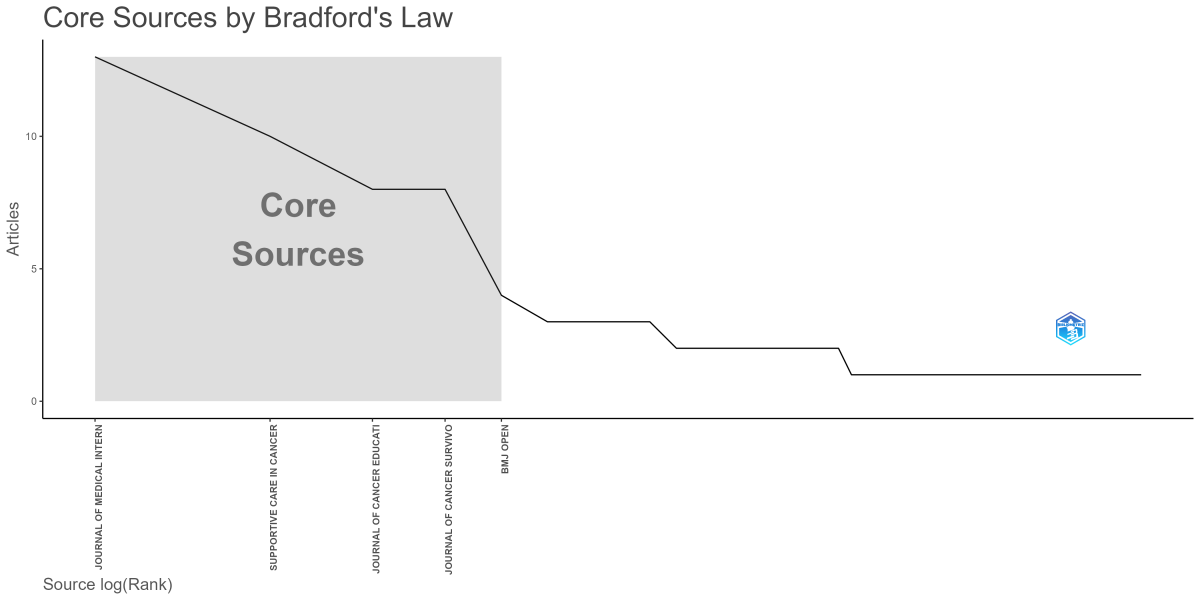

Supplement: Multimedia Appendix 2 [file cancer-v11-e77214-s002.png]

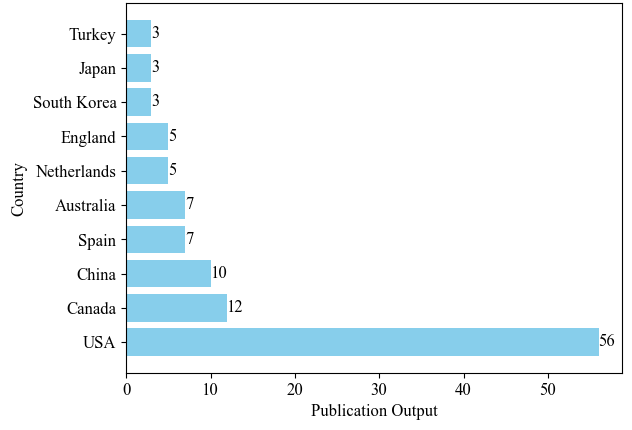

Supplement: Multimedia Appendix 3 [file cancer-v11-e77214-s003.png]
